# Condensed Form of Nitrous Oxide Gas

**Published:** 1871-09

**Authors:** 


*Condensed Form of Nitrous Oxide Gas.*—At the late meeting of the American Dental Association, held at the Greenbrier White Sulphur Springs, a condensed form of nitrous oxide gas with apparatus, was on exhibition, and the advantages it possesses over the more bulky apparatus, and troublesome way of preparing it by the common process, was apparent to all. The advantages which this condensed form possesses, are described in an article we take from a correspondent of Middletown, Conn., which was published in the *Hartfort Post:*

"Anything that tends to relieve human suffering or to widen the application of the means for its relief is worth public attention; and in these days of quack nostrums where money is only sought without rendering any just equivalent, every real improvement should be hailed with delight. From the time that Horace Wells applied the nitrous oxide gas to produce insensibility during surgical operations, to the present, various methods have been devised, and many substitues for this gas have been introduced, all of which have had more or less merit, but none which have been as safe as the gas which was originally used for this purpose. But the objection to this was its bulk, and the difficulty of its manufacture, so that any one who used it to any extent must be provided with the means, and must understand the method of its manufacture. In addition to this was the uncertainty as to its purity and freedom from noxious and unpleasant odor, but when well prepared it was acknowledged to be by far the most pleasant and safe agent for producing insensibility. In England, about two years ago, this gas was successfully reduced to a liquid, but in order to retain it in that state it had to be kept in iron bottles of enormous strength to prevent bursting. In this form it was palatable and was used to a considerable extent, and with great success, particularly in the late Franco-Prussian war. Various attempts have been made in this country to learn the method of its manufacture, and sums as high as $5,000 have been offered for even a set of apparatus for this purpose. But our English cousins seem determined to keep this good thing to themselves, and refused to part with the secret. It is, therefore, the more gratifying to learn that Johnston Brothers, 812 Broadway, New York, sons of Professor John Johnston, of Wesleyan University, and educated at this place, have succeeded in accomplishing the same result, and can furnish one hundred gallons of this gas condensed into a liquid in a bottle twelve inches long by three inches in diameter, so that it can be carried with the surgeon or dentist and used at the home of the patient as well as at the office; and we can assure our readers that the effects are truly wonderful. By this method, too, the absolute purity of the gas is excluded by the very nature of the operation. Its effects, too, are more rapid and pleasant than those of the gas prepared by the old method.

"We are informed that these bottles are subjected to a hydrostatic pressure of 5000 pounds to the square inch before being used for this purpose. From this fact alone some idea may be formed of the complicated and powerful character of the apparatus employed in the manufacture of this new article."

